# Supplementary material for: A conformable fractional finite difference method for modified mathematical modeling of SAR-CoV-2 (COVID-19) disease
Source: PLoS One. 2024 Oct 28;19(10):e0307707. doi: 10.1371/journal.pone.0307707 (PMC11515971; doi:10.1371/journal.pone.0307707)
Supplement: Attachment — Submitted filename: PONE-D-23-25335.pdf [file pone.0307707.s001.pdf]

Manuscript Number: PONE-D-23-25335

Manuscript Title: **A Conformable Fractional Finite Difference Method for Modified Mathematical Modeling of SAR-CoV-2 (COVID-19) Disease**

Review Result

In the present manuscript investigate modified and comprehensive mathematical model that captures the complex relationships between various population compartments, including susceptible, infected, exposed, recovered, vaccinated, and quarantined people. Using conformable derivatives, we provide a system of equations that precisely captures the complex interconnections inside the COVID-19 transmission.

This field also has a wide applications. Clearly organized in analytical as well as numerical part. The work of the paper is **good**. However, there are some comments to improve the quality of the paper which are given as follows:

1. Model formation is unclear, explain it well.
2. Explain novelty or key contributions of your work in the end of introduction part.
3. There are lot of grammatical error, some typos and punctuation problems are exist that should be checked and corrected throughout the paper.
4. What about the solution of system?
5. What is the main objective of the research presented in the abstract, and why is it important in the context of the COVID-19 pandemic?
6. How does the mathematical model described in the abstract account for the different population compartments, and what role do conformable derivatives play in this model?
7. What methods were used for performing calculations in this research, and why was MAPLE-2019 chosen for these calculations?
8. How you fix the initial values of the model?
9. In Introduction part, Start with mathematical modeling and add some important works related with recent development in mathematical modeling such as Biomedical Signal Processing and Control, 84, 2023, 104714; Results in Physics, 2023, 106489; Nonlinear Dynamics, 111 4879-4914, 2023; Symmetry, 15(4), 845, 2023; Optimal Control, Applications and Methods, (43)(3), 842-866, 2022; Nonlinear Dynamics, 109 31693187, 2022.
