# Supplementary material for: A conformable fractional finite difference method for modified mathematical modeling of SAR-CoV-2 (COVID-19) disease
Source: PLoS One. 2024 Oct 28;19(10):e0307707. doi: 10.1371/journal.pone.0307707 (PMC11515971; doi:10.1371/journal.pone.0307707)
Supplement: Attachment — Submitted filename: Response Letter plos 2nd.pdf [file pone.0307707.s003.pdf]

Response to reviewer's comments

Manuscript ID: PONE-D-23-25335

Journal Name: PLOS One

Dear Associate Editor,

Thank you for your useful comments on our manuscript. We have considered your editorial or reviewer's comments and made the following changes to our paper entitled " A Conformable Fractional Finite Difference Method for Modified Mathematical Modeling of SAR-CoV-2 (COVID-19) Disease". We have modified the manuscript accordingly, and detailed corrections are listed below point by point. All the changes are highlighted in green, blue, and red color for Referee 1, Referee 2, and Referee 3, respectively in the paper.

Comments from Reviewers and Answers

Reviewer: I

The authors' revisions have been duly noted. Consequently, I hereby accept the manuscript. Their diligent updates have enhanced its quality, addressing concerns and refining content. Their efforts reflect a commitment to scholarly excellence, ensuring the work meets publication standards. Acknowledging their dedication, I endorse the manuscript for inclusion. This decision underscores its significance and contribution to the field. I anticipate its impact on readers and its role in advancing knowledge. With confidence in its merit, I affirm my acceptance of the manuscript, appreciating the authors' contributions and the collective endeavor toward academic advancement.

Response: We're grateful to you for your positive feedback and acceptance of our revised manuscript.

Reviewer: II

The authors were able to address some comments in the first revision. In my opinion, the paper can be accepted after revision taking into consideration the following points.

1. An in-depth discussion in the paper is necessary to establish why the proposed model based on

conformable fractional derivative is better than classical models based on ODE systems. These need to be highlighted as well in the numerical results.

Response: We appreciate the reviewer's suggestion and agree that an in-depth discussion is crucial. In our revised manuscript, we will elaborate conformable fractional derivative is better than classical models based on ODE systems. We will also enhance our numerical results section 4 to include a comparative analysis demonstrating how our fractional derivative model provides improved accuracy and insight into system behaviours as the fractional parameter ϕ approaches 1, thereby bridging the understanding between classical and fractional models in the discussion section.

2. The paper claims that the use of a finite difference approach over classical numerical methods gives "high convergence", but the paper fails to satisfactorily establish this, both theoretically and numerically.

Response: Table 3 explains that the finite difference approach gives high convergence results.

3. There are still grammatical, typographical, punctuation, and typesetting issues. I urge the authors to hire a copyeditor to help address these issues.

Response: All grammatical, typographical, punctuation, and typesetting issues have been removed.

Reviewer: III

The authors have not addressed all my comments.

1. Graphical illustrations need explanations. Typos exist there.

Response:

Thank you for your kind suggestion. Graphical explanations have been added and remove all typos.

2. Still, the literature needs to be updated about the NSFD method. It is strongly recommended to put relevant references about the methodology used for COVID-19. Authors have ignored these. They must cite the research work relevant to COVID-19 recently available like: Study of a mathematical model of COVID-19 outbreak using some advanced analysis." Waves in Random

and Complex Media (2022): 1-18. Study of integer and fractional order COVID-19 mathematical model." Fractals (2023): 2340046.Fractional order modeling of predicting covid-19 with isolation and vaccination strategies in Morocco." CMES-Comput. Model. Eng. Sci 136 (2023): 1931-1950.On an SEIR-type model of COVID-19 using piecewise and stochastic differential operators undertaking management strategies." (2023).
For NSFD cite these work: Results in Physics 52 (2023): 106890, Results in Physics 24 (2021): 104069.

Update the literature about applications of mathy. models like: Statistical and computational analysis for corruption and poverty model using Caputo-type fractional differential equations." Heliyon (2024)., A Fractal-Fractional Order Model to Study Multiple Sclerosis: A Chronic Disease." Fractals (2024): 2440010. South African Journal of Chemical Engineering 48 (2024): 63-70.

Response: Thank you for your suggestions. All suitable references have been cited according to instructions.

Response to Associate Editor:

The authors would like to thank the referees for suggesting specific changes in the original manuscript, for their valuable comments which improved the paper and for their high interest in this work. The manuscript has been resubmitted to your journal. We look forward to your positive response.

With Regards,

Tamour Zubair
